# The Effects of Tyrosine Kinase Inhibitors (TKIs) in Monotherapy and with Add-on Treatments on Health-related Quality of Life of People with Chronic Myeloid Leukemia: A Systematic Review of Randomized-Controlled Trials

**DOI:** 10.2174/17450179-v17-e211118-2021-HT2-1910-12

**Published:** 2023-01-05

**Authors:** Antonio E. Nardi, Federica Sancassiani, Vanessa Barrui, Goce Kalcev, Veronica Uras, Giulia Meloni, Luigi Marongiu, Giorgio Tamburini, Alberto Maleci, Laiana A. Quagliato, Giorgio La Nasa, Mauro Giovanni Carta

**Affiliations:** 1 Institute of Psychiatry, Federal University of Rio de Janeiro, Rio de Janeiro, Brazil; 2 Department of Medical Sciences and Public Health, University of Cagliari, Cagliari, Italy

**Keywords:** Chronic myeloid leukemia, Hematology, Health related quality of life, Psycho-oncology, Psychosocial health, Systematic review

## Abstract

**Background:**

The era of establishing tyrosine kinase inhibitors (TKIs) in the treatment of chronic myeloid leukemia (CML) changed the outcome and the course of this life-threatening malignancy. People suffering from CML have now a better prognosis and a longer life expectancy due to the development of TKIs, even if it requires long-term, often lifelong, treatments that are nonetheless associated with improved Health-related Quality of life (HRQoL). However, data on the effects of TKIs on HRQoL are not always systematic; sometimes the data have been obtained by studies different from RCTs, or without a clear definition of what HRQoL is. The main purpose of this systematic review is to summarize all randomized-controlled trials (RCTs) including HRQoL as main or secondary outcome in patients with CML treated with TKIs or with TKIs plus an add-on treatment.

**Methods:**

A systematic review has been conducted by searching the relevant papers in PubMed/Medline and Web of Science with the following keywords: “quality of life” OR “health-related quality of life” OR “QoL” OR “HRQoL” OR “H-QoL” AND “chronic myeloid leukemia”. Interval was set from January 2000 to December 2020.

**Results:**

40 papers were identified through the search. Out of them, 7 RCTs were included. All the studies used standardized measures to assess HRQoL, even not always specific for CML. 5 RCTs randomized subjects to 2 or 3 arms to evaluate the effects of TKIs of the first, second and third generation in monotherapy. 2 RCTs randomized subjects to TKI therapy plus an add-on treatment *versus* TKI therapy as usual. The results of all these trials were examined and discussed.

**Conclusion:**

All the included RCTs pointed out significant findings regarding the positive effects of TKIs on HRQoL of people with CML, both when they were used in monotherapy or, notably, with an add-on treatment to enhance TKIs effects.

## 1 INTRODUCTION

### 1.1 Treatments for Chronic Myeloid Leukemia: State of the Art

Chronic myeloid leukemia (CML) is a cancer of hematopoietic stem cells. It has an incidence of 1-2 cases per 100.000 adults, accounting for almost 15% of newly diagnosed cases of leukemia in adults [1]. The main characteristic of this type of leukemia is the increased and unregulated growth of myeloid cells in the bone marrow and the accumulation of these cells in the bone marrow and peripheral blood. Myeloid, erythroid, monocytic, megakaryocytic, B-lymphoid, and occasionally T-lymphoid lineage are abnormal cells observed in CML [2]. According to this, the three stages in the natural history of CML are the chronic phase, the phase of acceleration, and the blast phase. Most patients are randomly diagnosed as a result of an elevated white blood cell level during the chronic aSymptomatic period. The most frequent Symptoms found in patients with CML involve weight loss, fatigue, abdominal fullness, splenomegaly, leukocytosis, bleeding, purpura, thrombocytosis, and anemia [2]. In addition to this, recommendations for the management of CML have been improved by an international team of experts [3], as well as the proposal for stopping the treatment with TKI in patients with a prolonged molecular remission [4]. Perception of the role of various agents and clinical hues between them in the treatment of CML is crucial to optimizing the clinical outcome of the patient.

The era of establishing tyrosine kinase inhibitors (TKIs) in the treatment of CML, since the introduction of the first-generation TKI imatinib, completely changed the outcome and the course of this life-threatening malignancy. Nilotinib belongs to the second generation of TKIs and plays an extremely crucial role in the treatment of CML. It is one of the three drugs in this category together with dasatinib and bosutinib [5]. Nilotinib can also be used after ineffective or intolerant treatment with two different TKIs, and it becomes a third-line drug by indication [6]. Finally, ponatinib, a third generation TKI, is effective in patients with mutations, even if burdened by cardiovascular side effects [7].

Nilotinib has a leading role in the current treatment of CML and has proved its efficacy as a drug in the first and second line. The adverse drug reactions (ADRs) are usual in CML clinical practice [8]. Side effects of second-generation TKIs can endanger people's lives and are often associated with vital organs, such as the cardiovascular system [9].

The occurrence of ADRs (*i.e.*, myelosuppression, rash, nausea, diarrhea, fatigue, musculoskeletal pain, arthralgia, myalgia end endocrine disfunction) [10], even if mild, negatively impacts on CML patients’ perceived HRQoL, as well as on adherence to TKIs [8, 11]. Patients who reported that ADRs had a negative impact on their daily HRQoL, particularly on mood, general condition, enjoyment of life, walking, relationships, and work in general, perceived more frequent ADRs than those who did not experience this negative impact [8].

Furthermore, most patients with CML are diagnosed in the chronic phase with relatively mild or no Symptoms [1], such that maintaining long-term optimal adherence to TKIs, as well as reducing ADRs, could be challenging to improve patients’ disease control, optimal molecular response [12] and their perceived HRQoL.

For these reasons, psychosocial-supportive add-on treatments, in combination with TKIs therapies, have been recently developed, and the literature is starting to show their efficacy to teach patients with CML new ways to think about and cope with TKIs’ ADRs, as well as to improve their adherence, also by the use of new technologies [13, 14]. These kinds of combinate treatments could have a positive impact also on CML patients’ perceived HRQoL [15].

### 1.2 HRQoL of Patients with Chronic Myeloid Leukemia

Since the 80s, the U.S. Food and Drug Administration (FDA) [16, 17] argues regarding the importance of the inclusion of HRQoL assessment, together with other patient-reported outcomes (PROs), along with traditional clinical outcomes in the evaluation of treatment effectiveness for people with cancer [18-22].

Particularly, people suffering from CML have now a better prognosis and a longer life expectancy due to the development of TKIs [23], even if it requires long-term,often lifelong, treatments that are nonetheless associated with improved HRQoL as reported by patients [24, 25]. Mainly for these reasons, HRQoL is a relevant outcome for people with CML [19, 25, 26].

HRQoL is a complex multidimensional construct that includes illness perception and self-evaluation of the physical, mental, and social health status of the individuals [27]. As pointed out by Karimi and Brazier [28], there are at least four definitions of HRQoL in the literature. The first one refers to how well a person functions in their life, and his/her perceived well-being in physical, mental, and social domains of health [29]. The second definition distinguishes between Quality of Life (QoL) and HRQoL, pointing out that, while QoL is an all-inclusive concept incorporating all factors that impact upon an individual’s life, HRQoL includes only those factors that are part of an individual’s health, without including, for example, economic and political aspects [30]. The third one does not differentiate HRQoL from QoL, such that HRQoL is the aspect of QoL most affected by a disease [27]. Finally, the fourth definition of HRQoL highlights the value of the self-perceived health status [30-32].

In the last decades, a research had been focused on HRQoL and the burden on this PRO due to several chronic diseases, such as Multiple Sclerosis [33], Celiac Disease [34], Fibromyalgia [35], Carotid Atherosclerosis [36], Wilson’s Disease [37], hematological cancers [38], and solid cancers [39].

The main purpose of this systematic review is to quantify, describe and summarize all randomized-controlled trials (RCTs) that have included HRQoL as main or secondary outcome in patients with CML treated with TKIs or with TKIs plus an add-on treatment to enhance their effects and the patients’ endurance, as well their coping strategies for the ADRs.

## 2 METHODS

Recommendations from the Preferred Reporting Items for Systematic Reviews and Meta-analyses (PRISMA) guidelines were used in the mapping, organization, and implementation of the protocols to conduct the search strategy [40].

### 2.1 Search Strategy

Databases including PubMed/Medline and Web of Science were searched from January 2000 to December 2020. The search queried the following keywords: “quality of life” OR “health-related quality of life” OR “QoL” OR “HRQoL” OR “H-QoL” AND “chronic myeloid leukemia”. Furthermore, the references of all articles identified were carefully searched for additional studies. We reviewed all papers published in the English language.

### 2.2 Inclusion and Exclusion Criteria

Studies were included if they met the following criteria: (a) they enrolled people with Chronic Myeloid Leukemia (CML), (b) a Tyrosine Kinase Inhibitors was administered; (c) there was a placebo comparison group; (d) the impact of Tyrosine Kinase Inhibitors (TKIs) on HRQoL of patients with CML was reported; and (e) the study design was a randomized controlled trial.

Any study other than randomized controlled trials, duplicate studies, studies with data analysis still pending, studies evaluating the effects on HRQoL by using drugs different from TKIs and those not written in English were excluded.

### 2.3 Data Extraction and Analysis

Data on study-, patient-, and treatment-related characteristics were abstracted onto a standardized form. The following variables were extracted from all studies: authors, year of publication, country, overall sample size, effects on HRQoL, and drugs measure characteristics (duration of treatment and dose of drugs).

The critical qualitative appraisal of the systematic review was done by the Scottish Intercollegiate Guidelines Network (SIGN) implementation of the assessment of Methodology Checklist 2: Controlled Trials. Two evaluators independently assigned the rate to each item of the evaluation scale (Table **1**).

### 2.4 Data Synthesis and Presentation

We summarized the main findings with a focus on the outcome of interest (Table **2**).

## 3 RESULTS

The literature search identified 39 potentially relevant articles for initial screening. One additional paper was recognized through other sources. Duplications (n=27) were identified by manual screening of the titles. Full texts were obtained on 12 abstracts that were classified as possible for inclusion. Finally, 7 papers were included in the qualitative synthesis, after the exclusion of 3 studies because they were focused on the effects on HRQoL by drugs different from TKIs, and 2 papers considered one arm treated with a TKI and the other with a different kind of drug. The process of inclusion/exclusion of studies is summarized in Fig. (**1**) with the help of a PRISMA Flow Diagram.

### 3.1 General Characteristics of the Included Studies

The qualitative synthesis included 7 RCTs involving 1.833 subjects with CML in active treatment by TKIs. The papers were published between 2014 and 2020 (Table **2**).

Out of them, 3 were multicenter RCTs studies, while 2 were conducted in the same cohorts across the US and EU [41]. The first one [42] considered several Patient-Reported Outcomes (PROs), and HRQoL was one of them; the second one [43] was focused on the association between the molecular response (MR) to TKIs and HRQoL. N 1 was a multicenter RCT in the US [44]. N 1 RCT was conducted in China [45], N 1 RCT in Malaysia [46] and N 2 RCTs were carried out in the US [47, 48].

HRQoL was considered as the primary outcome in N 2 RCTs [45, 47]; in N 1 RCT, HRQoL was indicated as the primary outcome in a post-hoc analysis [44]. In N 1 RCT [43], it was indicated as the primary outcome in association with the molecular response (MR). In N 3 RCTs [42, 46, 48], HRQoL was considered a secondary outcome.

### 3.2 Kind of the Interventions Implemented

In the included studies, subjects were randomized to receive: Imatinib 400 mg/daily or Bosutinib 400 mg/daily treatments for 5 years [42, 43]; Dasatinib 100 mg/day or Nilotinib 400 mg twice/daily or Ponatinib 30-45 mg/daily treatments for 2 years [47]; Imatinib 400 mg/daily or Nilotinib 300 mg twice/daily for 5 years [45]; Nilotinib 300 mg twice/daily or Nilotinib 400 mg twice/daily or Imatinib 400 mg/daily for 2 years [44]; TKI therapy + internet-assisted Cognitive-Behavioral Therapy targeted therapy-related fatigue (CBT-TTF) or TKI therapy + waiting list control (WLC) for 18 weeks [48]; TKI therapy + medication management service-MMS or TKI therapy + usual care for 1 year [46]. The last N 2 RCTs [46, 48] used an add-on treatment for TKI therapy to enhance its effects.

### 3.3 Instruments to Assess HRQoL

As shown in Table **3**, all the included RCTs were conducted by using validated instruments to assess HRQoL.

Particularly, in N 3 RCTs [42-44], the FACT-leu was used [49]. This questionnaire includes a series of general HRQoL items (FACT-General [FACT-G]) and a leukemia-specific subscale referred to the 7-day period preceding the survey administration. Each item is scored on a scale from 0 (not at all) to 4 (very much), with higher scores indicating better HRQoL. The 27-item FACT-G (score range 0–108) is referred to 4 domains: physical well-being (PWB) (7 item; score range 0–28), social well-being (SWB) (7 items; score range 0–28), emotional well-being (EWB) (6 items; score range 0–24), and functional well-being (FWB) (7 item; score range 0–28). The 17-item leukemia-specific subscale (score range 0–68) evaluates patient knowledge about leukemia. The FACT-Leu total score (44 item; range 0–176) results from the sum of the FACT-G total score and the leukemia-specific subscale score. There is also an index, the Trial Outcome Index (TOI) FACT-Leu score (31 item; range 0–124), resulting from the sum of physical and functional well-being domain scores plus the leukemia specific subscale score.

In N 1 RCT [48], only the total score (range 0-108) of the Functional Assessment of Cancer Therapy–General (FACT-G) scale from the FACT-leu [49] was used, considering its 4 domains as described above (PWB, SWB, EWB, FWB).

The EuroQoL-5Dimension (EQ-5D) [50, 51] was used in N 1 RCT [39]. This questionnaire includes five items (mobility, self-care, usual activities, pain/discomfort, and anxiety/depression) rated on a Likert scale (no problems, some problems, or extreme problems), with higher levels indicating greater impairment. The EQ-5D utility score is a weighted health-state index score (range 0–1) based on patient responses to the five items in comparison with population-assessed weights to each set of responses. The EQ-5D also assesses daily patient-rated health-state on a visual analog scale (VAS), graduated (0–100). For the EQ-5D utility score and VAS, higher scores indicate better functioning.

In N 1 RCT [47], the MD Anderson Symptom Inventory (MDASI-CML) was used [49]. By this questionnaire, the severity of 20 Symptoms of patients is evaluated: 13 cancer-related Symptoms (fatigue, difficulty remembering/memory, nausea, disturbed sleep, vomiting, distress, pain, dyspnea, appetite, drowsiness, dry mouth, numbness, and sadness) plus 7 CLM-specic Symptoms (muscle soreness/myalgia, swelling of extremities, bruising/ bleed, skin rash, malaise, headache, and diarrhea). The questionnaire also includes 6 items referring to interference with various aspects of HRQoL by 2 indexes: WAW (Work, general Activity, Walking), and REM (Relations with others, Enjoyment of life, Mood). All the items are scored from 0 (not present) to 10 (worst) and classified as mild (scores 1-4), moderate (scores 5-6), or severe (scores 7-10) interference.

The Short Form Health Survey - 36 item (SF-36) [52, 53] was used in N 2 included RCTs [44, 45]. This self-report questionnaire is an HRQoL measure consisting of 36 items composing 8 dimensions: physical functioning (PF), role limitation due to physical health problems (RP), bodily pain (BP), general health perceptions (GH), vitality (VT), social functioning (SF), role limitations due to emotional problems (RE), and mental health (1). The 8 subscales are grouped into 2 indexes: the physical component summary (PCS) and the mental component summary (MCS), each one ranging from 0 to 100, with higher scores indicating better HRQoL in the 28-day period preceding the survey administration.

In N 1 included RCT [46], the EORTC_QLQ30_CML24 [54] was used. The EORTC QLQ-30_CML-24 is a self-report questionnaire developed to assess HRQOL among people suffering from CML. Specifically, the EORTC core quality of life 30-item questionnaire (QLQ-30) includes 5 functioning scales (physical, role, emotional, cognitive, and social); 3 Symptoms scales (fatigue, nausea/vomiting, and pain); 6 single-item scales (dyspnea, insomnia, appetite loss, constipation, diarrhea, and financial impact); and the global health status/QoL scale. The EORTC supplementary chronic myeloid leukemia 24-item questionnaire (CML-24) consists of 6 scales: impact on daily life, Symptom burden, impact on worry/mood, body image problems, satisfaction with care and information, as well as satisfaction with social life. Higher scores indicate worse HRQoL.

### 3.4 Findings by the Included RCTs with respect to HRQoL

In the study conducted by Cortes *et al*. [42], the assessment of HRQoL in people with CML was an exploratory objective. Two measures were used to assess HRQoL: the FACT-leu [46] and the EuroQoL-5Dimension (EQ-5D) [50, 51]. It was found that, at the baseline, mean scores for FACT-G domains, FACT-G total, FACT-Leu total, and TOI FACT-Leu were similar in the two treatment arms. All FACT-Leu scores pointed out HRQoL’s improvement or maintenance with bosutinib or imatinib treatment after one year from the baseline. Improvements at month 12 for functional well-being, FACT-G total, FACT-Leu total, and TOI FACT-Leu were significant in the imatinib but not in the bosutinib arm. Regarding the minimal important difference (MID) in FACT-Leu scores, there were no clinically meaningful improvements in either treatment arm. Minimal changes from the baseline were noted with both bosutinib and imatinib treatments in physical well-being and social well-being scores, confirming the maintenance of these HRQoL domains. Repeated-measures mixed effects models did not point out any significant difference between bosutinib and imatinib for any FACT-Leu subscale from the baseline to one year. Regarding EQ-5D utility and VAS mean scores at baseline, they were similar in the bosutinib and imatinib arms. During the treatment, EQ-5D utility and VAS mean scores improved or were maintained in both treatment arms. Particularly, after one year, a significant improvement in EQ-5D VAS mean score was observed with both bosutinib and imatinib; a significant improvement in the EQ-5D utility score was observed in the imatinib arm only. The number of patients reporting no, some, or extreme problems in each of the EQ-5D items remained relatively constant in the bosutinib arm. In the imatinib arm, the proportion of patients reporting no problems with mobility, usual activities, or pain/discomfort increased after treatment, even if the percentages for self-care and anxiety/depression remained relatively stable. Repeated-measures mixed-effects models for all EQ-5D scores pointed out any difference between bosutinib and imatinib arms regarding mobility after one year. The authors concluded that their findings show that, in addition to experiencing improved clinical outcomes in comparison with imatinib, patients with CML receiving bosutinib can maintain or improve their HRQoL during treatment, with relatively low Symptom burden and drug tolerability.

The study carried out by Brümmendorf *et al*. [43], as well as that of Cortes *et al*. [42] just described above, regard findings from BFORE trial [41], a multicenter study primarily aimed to assess the efficacy and safety of bosutinib *versus* imatinib for first-line treatment of chronic-phase CML. Brümmendorf *et al*. [43] examined the relationships between molecular response (MR) and HRQoL after imatinib or bosutinib treatments. They represented MR values by a Log Reduction scale (MRLR), in order to consider the MRLR as a continuous variable. A repeated-measures longitudinal model was used to estimate the relationships between the MRLR as a predictor and each FACT-Leu domain [46] as an outcome with respect to HRQoL. Furthermore, the authors established the minimal important difference (MID) as a change clinically meaningful to a patient for physical well-being, emotional well-being, functional well-being, leukemia-specific, FACT-G, FACT-Leu total, and for TOI-FACT-Leu scores. The MID for social well-being has not been defined. Finally, the differences in estimated mean FACT-Leu domain and aggregated domain scores corresponding to MRLR values of − 5 (MR5), − 3 major MR (MMR), and − 1 (MR1) were compared with MRLR value of 0 (standardized baseline; no response). Findings pointed out that FACT-Leu total score differences corresponding to MR5, MMR, and MR1 were significant (p < 0.0001). Differences corresponding to MR5, MMR, and MR1 were significant (p < 0.05) for FACT-G total, emotional well-being, functional well-being, leukemia-specific, and TOI-FACT-Leu scores, but were not significant for physical or social well-being scores. Only patients who achieved a deep MR (MR5) exceeded the MID for the FACT-Leu total, FACT-G total, and TOI-FACT-Leu scores. The MID was not reached for physical well-being, emotional well-being, functional well-being, or leukemia-specific scores, regardless of the depth of response. The authors concluded that there was a variable impact of clinical improvement in different dimensions of HRQoL in patients with CML. Overall, a better response to TKIs treatment is associated with improving HRQoL. For patients with deep MR, emotional well-being and leukemia-specific domains showed the greatest improvement, whereas social well-being and physical well-being FACT-leu domains exhibited the weakest relationships with MR.

The study carried out Zulbaran Rojas *et al*. [47] aimed to investigate the Symptom burden associated with TKIs therapy (dasatinib or nilotinib or ponatinib) and its impact on HRQoL and treatment outcomes. Patients were assessed with the MDASI-CML at the specified intervals. Overall, fatigue exhibited the highest mean scores for all three cohorts. Regarding interference with daily activities, WAW (Work, general Activity, Walking) was the most impacted variable. There was no significant difference for fatigue mean scores between the three cohorts. Patients treated with ponatinib showed significantly higher scores on disturbed sleep than patients on dasatinib. Furthermore, they showed higher scores on malaise, swelling of extremities, pain, shortness of breath and WAW than patients treated with nilotinib. Finally, patients treated with ponatinib showed significantly higher skin rash, muscle cramps, dry mouth, distress and REM (Relations with others, Enjoyment of life, Mood) in comparison to both dasatinib and nilotinib cohorts. Patients treated with nilotinib had significantly higher scores of disturbed sleeping, diarrhea and swelling of extremities than patients on dasatinib. Patients on dasatinib showed significantly higher scores in comparison with those on nilotinib. Finally, the majority of patients experienced persistent mild Symptoms at each time assessment point (3, 6, 9, 12, 18, and 24 months from the start of the therapy). Regarding the association between the molecular response and the Symptoms that impact on HRQoL, fatigue, disturbed sleep, and drowsiness were the most severe Symptoms that coincide in the longitudinal analysis for both the overall population and when analyzing each cohort separately. However, overall patients achieving deep molecular response after 1 year showed better scores regarding Symptomatology in comparison with patients not achieving it. This held true also for interference with activities of daily life. Even though patients with a non-optimal MR had a worse mean Symptoms score, there was no significant difference between the majority of Symptoms. Furthermore, their mean Symptoms scores showed a downward trend over time, whether patients achieving a complete MR did not. The authors concluded that patients treated with TKIs show chronic, mild-moderate adverse events that affect their HRQoL and interfere with daily activities. The severity peaked between 6 and 9 months from the start of therapy and improved gradually over time but tended to remain present up to 24 months after the start of therapy.

The RCT by Yu *et al*. [45] aimed to assess the relationship between molecular responses (MR) within 1 year and HRQoL by exploring profiles of patients with CML who were treated with imatinib or nilotinib. The authors used the Short Form Health Survey – 36 items (SF-36) [53] to assess the HRQoL. Using a multivariate analysis, findings showed that achievement of optimal MR at 6 months was associated with a tendency of having high physical functioning (PF), social functioning (SF), and role limitations due to emotional problems (RE), while achieving optimal response at 12 months was associated with markedly higher role limitation due to physical health problems (RP) and RE. Furthermore, age < 40 years was associated with better PF, better Physical dimension of HRQoL, SF and RE; female gender was associated with better SF and RE; and a higher level of education was associated with better bodily pain (BP). MR at 3 months and the TKI used (imatinib or nilotinib) did not show any impact on the HRQoL during TKIs therapy. By using another multivariate analysis, the authors assessed the associations between factors, including treatment responses, patient characteristics, TKI used, duration of therapy and the longitudinal change in the HRQoL profile in patients undergoing TKIs therapy. Findings showed that the Physical dimension of HRQoL was stable over the treatment period, while the Mental dimension of HRQoL showed a tendency toward a gradual increase; however, achieving optimal MR at 12 months was the sole factor associated with a significant improvement in the Physical dimension of HRQoL over time. The authors concluded that the use of TKIs (imatinib or nilotinib) did not show any impact on HRQoL during the treatment. Each subscale of HRQoL at the baseline between patients achieving or not optimal MR at 3, 6, or 12 months during TKIs therapy was similar. Achieving optimal MR by 12 months of treatment was associated with longer survival, lower rates of treatment failure and disease progression but also with better HRQoL in patients with CML treated with TKIs.

The study of Guérin *et al*. [44] aimed to identify the low-grade adverse events (AEs) due to TKIs use which significantly impact the HRQoL of patients with CML. The authors used two instruments to assess the HRQoL: the FACT-leu [49] and the Short Form Health Survey – 36 items (SF-36) [53]. AEs were grouped in accordance with the MedDRA System Organ Class (SOC) classification [55], where they are separated into 26 categories based on etiology (*i.e.*, infections and infestations), manifestation site (*i.e.*, gastrointestinal disorders) or purpose (*i.e.*, surgical and medical procedures). AEs were grouped based on their severity level, according to the Common Terminology Criteria for Adverse Events (CTCAE) Version 3.0.28. For the purposes of their study, low-grade AEs were defined as AEs with CTCAE grades 1 (mild) and 2 (moderate), and high-grade AEs were defined as AEs with CTCAE grades 3 (severe) and 4 (life-threatening or disabling). Regarding the FACT-Leu assessments, 58.5% showed an improvement from the baseline, 39.9% a deterioration from the baseline, and 1.6% showed a relative stability from the baseline. The percentages for SF-36 Physical dimension were 53.5% for patients who improved from the baseline, 46.1% for patients who showed a deterioration and 0.4% for those who showed a relative stability from the baseline. Regarding SF-36 Mental dimension, patients who improved from the baseline were 63.1%, those who showed a deterioration were 36.5% and patients who remained stable were 0.4%. Overall, 250 (9.7%) of the FACT-Leu assessments and 747 (30.8%) of the SF-36 assessments were preceded by a low-grade AE during their corresponding 7- and 28-day recall periods. Gastrointestinal (GI) Symptoms were the most common AEs that were recorded in the recall period for both FACT-Leu and SF-36. Four SOC categories of low-grade AEs had a prevalence of ≥1% during the 7-day recall period of the FACT-Leu assessments and 13 SOC categories had a prevalence of ≥1% during the 28-day recall period of the SF-36 assessments. After adjusting for potential confounders, the following low-grade AEs were found to significantly negatively impact patients’ HRQoL based on at least one of the three HRQoL scores: gastrointestinal (GI) Symptoms for FACT-Leu and SF-36 Physical dimension, blood and lymphatic system Symptoms for SF-36-Physical dimension, general disorders and administration site conditions (*i.e.*, fatigue, peripheral edema) for SF-36 Mental dimension, musculoskeletal Symptoms for SF-36 Physical dimension, and psychiatric Symptoms (*i.e.*, insomnia, anxiety) for SF-36 Mental dimension. The authors also considered the differences in the incidence of these low-grade AEs between the three treatment arms. GI, blood and lymphatic system, musculoskeletal, general disorders and administration site conditions had a lower incidence in the nilotinib arms than in the imatinib arm. The incidence of the psychiatric Symptoms was similar between the three treatment arms. These trends did not change after adjustment for potential confounders. The authors concluded that the impact of low-grade AEs on HRQoL should be taken into account, along with other factors, when selecting the optimal treatment for people with CML.

The study carried out by Jim *et al*. [48] aimed to conduct a pilot RCT of internet-assisted cognitive behavioral therapy for targeted therapy–related fatigue (CBT-TTF) in comparison with a waitlist control (WLC) in patients with CML treated with a TKI that reported fatigue as a side effect of TKI therapy. The authors hypothesized that the CBT-TTF group would show significantly improved fatigue (primary outcome) and HRQoL (secondary outcome) in comparison with the WLC group. The CBT-TTF intervention was conducted over 18 weeks and consisted of a 90 minutes initial vis-a-vis session at the cancer center to introduce the intervention and its rationale and outline the tailored treatment plan. Subsequent sessions were conducted by FaceTime call for the iPad, lasted 45 minutes and followed a basic format of problem recognition, solution generation, implementation, and progress evaluation. Depending on participants’ progress in meeting therapy goals, the sessions’ schedule was at 1- or 2-week intervals. A final session summarized progress and ways to maintain therapeutic gains. The HRQoL was measured by the total score of the Functional Assessment of Cancer Therapy–General (FACT-G) scale [49] and its 4 subscales: Physical Well-Being (PWB), Functional Well-Being (FWB), Emotional Well-Being (EWB), and Social Well-Being (SWB). Regarding the TKIs therapies undergone by the patients who participated in the trial (N 44), they had been on their current TKI for 2.5 years; 36% of patients were receiving dasatinib, 25% nilotinib, 20% bosutinib, 12% imatinib and 7% ponatinib with no difference in proportions between the two arms. Findings regarding HRQoL showed significantly greater improvements in the CBT-TTF group for overall HRQoL (p=0.005). The effect size for HRQoL was large (d = 1.15). More CBT-TTF participants (88%) reported clinically significant improvements in HRQoL than WLC participants (54%; p<0.016). Also, with respect to the H-QoL dimensions, significant improvements were found in the CBT-TTF group than the WLC group for FWB (d=1.06; p=0.003) and EWB (d=1.12; p<0.001) but not for PWB (d=0.45; p=0.151) or SWB (d=0.60; p=0.800). The authors concluded that the acceptability and feasibility of the intervention depend mainly on its remote delivery by FaceTime for the iPad. Many patients lived far away from the cancer service and/or hold a full-time job, such that they could not attend a vis-a-vis intervention; furthermore, they highlighted that delivery of CBT-TTF is consistent with the increasing use of telemedicine in cancer care.

The study by Tan *et al*. [46] aimed to evaluate the impact of a medication management service (MMS) on adherence to TKIs (primary outcome), molecular responses (secondary outcome) and HRQoL (tertiary outcome) in patients with CML. Regarding the TKIs therapies undergone by the patients who participated in the trial (N 129), 50.8%, 47.7%, 0%, and 1.5% of the patients in the intervention arm and 56.3%, 42.2%, 1.5%, and 0% in the control arm received imatinib, nilotinib, dasatinib and ponatinib, respectively, with no difference in proportions between the two arms. The MMS intervention (add-on to TKIs therapies) consisted of five pharmacy service events over a 6-month period. These included three face-to-face interactions (at recruitment, 3rd and 6th month) and two telephone follow-ups with a pharmacist at 1st and 4th month after the recruitment. The face-to-face interactive sessions were delivered by an iPad and a printed information booklet, containing information regarding CML, goals of TKI therapy and the importance of adhering to therapy. The participants also received two adherence aids: TKI blister packaging labeled with calendar dates, and installation of smartphone medication adherence reminder application, MediSafe. At the first follow-up (1st month after the recruitment), participants in the MMS arm were contacted by telephone to explore the feasibility of the initially proposed intervention, and if needed, alternative strategies were introduced to help patients to manage any persistent drug-related problems (DRPs). A second study visit was scheduled at the 3rd month, corresponding with their regular clinic visit, where the participants were educated on possible adverse events of TKIs and to use adaptive coping strategies for adverse events. This was followed by a telephone call a month later (4th month). The third study visit was scheduled at the 6th month to update the medication list as needed, identify potential drug-drug, drug-herb, drug supplement and drug-food interactions, and to advise the participant on the management of these potential interactions. In this study, the instrument used to assess HRQoL was the European Organization for Research and Treatment of Cancer (EORTC)_QLQ-30_CML-24 [54]. Findings showed that, at the 6th month after the baseline, participants in the MMS arm reported a significantly better HRQoL than those in the control arm in the three subscales of EORTC QLQ-30_CML24, including financial difficulties, satisfaction with care and information, and satisfaction with social life. At the 12th month after the baseline, the other three subscales, including cognitive functioning, insomnia and impact on worry and mood, were also significantly improved in the MMS arm. Furthermore, eleven participants developed comorbidities or had surgery during the study period but none of the HRQoL dimensions was associated with the development of these comorbidities except for pain. The authors concluded that the MMS significantly improved adherence to TKIs and improved clinical outcomes, such as HRQoL of people affected by CML, since the efficacy of TKIs treatment also depends on supporting patients’ engagement, knowledge and commitment towards their treatment plans and medication regimen to achieve the best possible clinical outcomes.

## 4 DISCUSSION

This systematic review reports the main features and findings of RCTs aimed to evaluate the efficacy of TKIs in monotherapy or with add-on treatments to improve the HRQoL of people affected by CML.

All the studies included openly highlighted the need to assess the HRQoL of people with CML in order to link these data to the other clinical outcomes usually considered, such as the molecular response (MR) to TKIs treatment. Collectively, these findings build on the results reported by other systematic reviews examining the impact of TKIs therapy on HRQoL, as well as other patient-reported outcomes (PROs) of people with CML [24, 54, 57].

This systematic review differs in emphasis from previous research syntheses for at least three reasons. Firstly, because it includes exclusively RCTs aimed to consider the effects of TKIs therapies specifically on the HRQoL as a primary or secondary, or a tertiary outcome. Secondly, because it also includes some RCTs in which an add-on treatment for TKIs therapy showed an impact on HRQoL by enhancing the TKIs therapy effects on this patient-reported outcome. Finally, its focus is on patients with CML receiving prolonged (even possibly lifelong) TIKs treatment, such that pointing out the effects of TKIs therapies on patients’ HRQoL is crucial to more robustly inform patient care and improve healthcare quality [56].

The US Food and Drug Administration (FDA) [17] has proposed the expression “patient-reported outcomes” (PROs), which can be considered an umbrella term including several different constructs referring to any aspect of a patient’s health status that comes directly from the patient, emphasizing his/her unique experience about health and illness. Among the PROs, HRQoL could be considered the most deeply embracing concept involving many dimensions of a person's daily life. As the World Health Organization pointed out [54], HRQoL is not necessarily linked to a disease, such that it is an outcome used also in the general population to explore illness perception, as well as self-evaluation of physical, mental, and social health status [58, 59]. In the last century, research is moving away from a perspective focused just on negative indicators of disease and illness, looking for more positive indicators of mental and physical wellbeing to supplement the latter [59], as well as to improve health-costs containment, by emphasizing the concept that high-value care is a way to control spending without compromising quality; the benefits provided by different treatments should thus include the assessment of perceived HRQoL by the patients in order to determine the impact of care on their lives and to choose the best cost-effective treatment [60].

However, while there exist substantial comparative data on the efficacy and safety of newer TKIs in the hematology field, little is known regarding HRQoL as perceived by patients with CML treated with TKIs [61]. As stated by Brümmendorf *et al*. [43], the associations between the efficacy of treatment for CML by TKIs and HRQoL are largely unknown. In the present systematic review, out of the included seven, just two RCTs [45, 47] selected HRQoL as the primary outcome, and the other two considered it as a primary outcome in association with molecular response to the TKIs therapy [40] or in a post-hoc analysis [44], after considering other clinical outcomes from the original ENEST trial [62].

All the included studies pointed out significant findings related to the positive effects of TKIs on HRQoL, also in terms of minimal important difference (MID) when the FACT-LEU was administered, both when they were used in monotherapy or with an add-on treatment. Generally, the MID, a change that is clinically meaningful to a patient, has been defined as 2–3 points for physical well-being, 2 points for emotional well-being, 2–3 points for functional well-being, 4–7 points for leukemia-specific, 3–7 points for FACT-G, 6–12 points for the FACT-Leu total, and 5–6 points for TOI-FACT-Leu scores; the MID for social well-being has not been defined [63], as well as for the dimensions assessed by other tools not specific to assess HRQoL in CML.

It is notable that recent RCTs in the field of CML care increasingly aim to evaluate the effects of TKIs plus an add-on treatment able to enhance their effects on several PROs [13-15]. Particularly, this systematic review selected those that considered HRQoL specifically as an outcome among the other PROs [46, 48]. Furthermore, it is of interest that the add-on treatments proposed in these RCTs are focused on the use of new technologies and telemedicine (an internet-assisted cognitive behavioral therapy delivered by FaceTime calls for the iPad [46] and a medication management service delivered by vis-à-vis and phone consultations with a pharmacist, as well by a mobile app among the other aids [48]). It is notable that the authors of both studies insist on several important issues that should be considered for further research, such as on the success of TKIs treatments that depends on patients with CML engagement, knowledge and commitment towards their treatment plans and medication regimen, the cost-effectiveness and the access for all of such add-on treatments in the routine clinical practice, or on the generalizability of the findings with larger and representative samples.

Finally, even if all the included studies used validated instruments to assess HRQoL, there is a relatively high heterogeneity in the kind of the instruments; not all of them are specific to evaluate HRQoL in people with CML, and not all RCTs consider the same HRQoL dimensions of the instrument chosen. Furthermore, the included RCTs are not always focused on the comparison of different classes of TKIs (*i.e.*, first *versus* second generation) with regard to the effects on HRQoL, or they randomize subjects to different arms of treatments characterized by a miscellanea of TIKs plus an add-on treatment to enhance TKIs’ effects on HRQoL. Considering this, it is not easy to provide a meta-analysis of the findings.

This systematic review has some limitations. Firstly, it was performed by searching in the Pubmed/Medline and Web of Sciences databases only. Other databases, such as PsycInfo/Ovid, could provide more findings related to RCTs. Finally, more RCTs could be found by searching among the references in the excluded papers, reviews and/or meta-analyses.

## CONCLUSION

All the included RCTs pointed out significant findings regarding the positive effects of TKIs on HRQoL of people with CML, both when they were used in monotherapy or, notably, with an add-on treatment to enhance TKIs effects. Even if all of them used validated instruments to assess HRQoL, there was much heterogeneity observed in the findings, which made it difficult to perform a meta-analysis.

## Figures and Tables

**Fig. (1):**
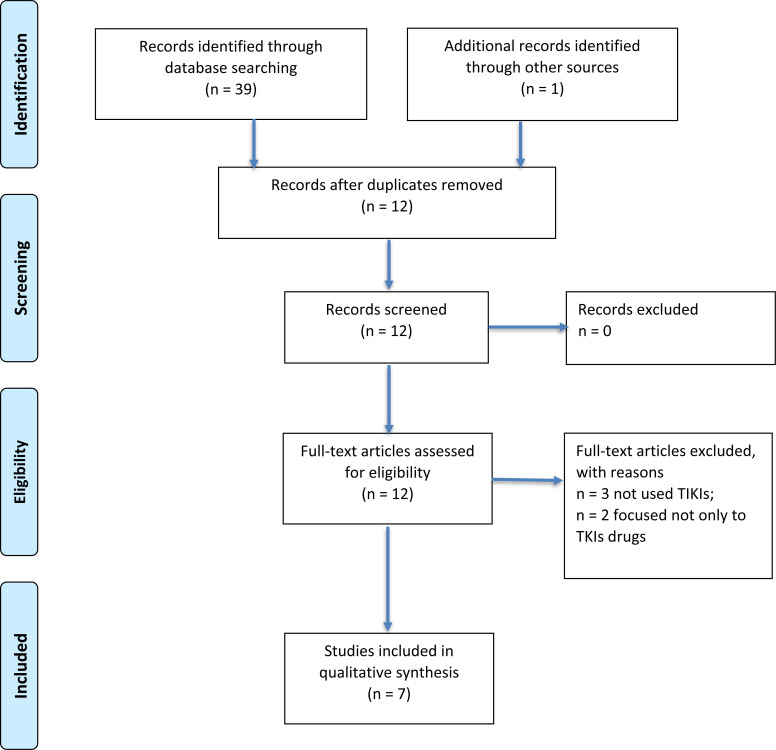
PRISMA 2009 flow diagram. **Adapted from:** Moher D, Liberati A, Tetzlaff J, Altman DG, The PRISMA Group (2009). Preferred Reporting Items for Systematic Reviews and Meta-Analyses: The PRISMA Statement. PLoS Med 6(7): e1000097. doi:10.1371/journal.pmed1000097. For more information, visit: http://www.consort-statement.org / www.prisma-statement.org

**Table 1:** Qualitative appraisal of the included studies by the Scottish Intercollegiate Guidelines Network (SIGN) - Methodology Checklist 2: Controlled Trials.

**Study identification** (Include author, title, year of publication, journal title, pages)	Yu *et al*. 2018 Achieving optimal response at 12 months is associated with a better health-related quality of life in patients with chronic myeloid leukemia: a prospective, longitudinal, single center study. BMC Cancer. 2018 Aug 3;18(1):782. doi: 10.1186/s12885-018-4699-5. PMID: 30075760; PMCID: PMC6091091.	Brümmendorf *et al*. 2020 Relationship between molecular response and quality of life with bosutinib or imatinib for chronic myeloid leukemia. Ann Hematol. 2020 Jun;99(6):1241-1249. doi: 10.1007/s00277-020-04018-1. Epub 2020 Apr 19. PMID: 32307568; PMCID: PMC7237399.	Tan *et al*. 2019 Efficacy of a medication management service in improving adherence to tyrosine kinase inhibitors and clinical outcomes of patients with chronic myeloid leukaemia: a randomised controlled trial. Support Care Cancer. 2020 Jul;28(7):3237-3247. doi: 10.1007/s00520-019-05133-0. Epub 2019 Nov 16. Erratum in: Support Care Cancer. 2019 Dec 7;: PMID: 31734798.	Zulbaran-Rojas *et al*. 2018 A prospective analysis of Symptom burden or patients with chronic myeloid leukemia in chronic phase treated with frontline second-and third-generation tyrosine kinase inhibitors. Cancer medicine. 2018; 7(11); 5457–5469. doi:10.1002/cam4.1808 Epub 2018 Oct 14. PMID: 30318751.	Cortes JE *et al*. 2019 Patient-reported outcomes in the phase 3 BFORE trial of bosutinib *versus* imatinib for newly diagnosed chronic phase chronic myeloid leukemia. J Cancer Res Clin Oncol. 2019;145(6):1589-1599. doi: 10.1007/s00432-019-02894-3. Epub 2019 Apr 15. PMID: 30989330.	Jim *et al*. 2020 Internet-assisted cognitive behavioral intervention for targeted therapy-related fatiguein chronic myeloid leukemia: Results from a pilot randomized trial. Cancer, 2020; 126(1):174-180. doi: 10.1002/cncr.32521. Epub 2019 Sep 25. PMID: 31553815.	Guérin *et al*. 2014 Impact of low-grade adverse events on health-related quality of life in adult patients receiving imatinib or nilotinib for newly diagnosed Philadelphia chromosome positive chronic myelogenous leukemia in chronic phase. Curr Med Res Opin. 2014;30(11):2317-2328. doi:10.1185/03007995.2014.944973. Epub 2014 Aug 5. PMID: 25025755.
**Guideline topic**	RCT_chronic myeloid leukemia_HRQoL	RCT_chronic myeloid leukemia_HRQoL	RCT_chronic myeloid leukemia_HRQoL	RCT_chronic myeloid leukemia_HRQoL	RCT_chronic myeloid leukemia_HRQoL	RCT_chronic myeloid leukemia_HRQoL	RCT_chronic myeloid leukemia_HRQoL
**Keywords no**	5	5	6	4	5	5	5
**Reviewers**	FS/GK	FS/GK	FS/GK	GK/FS	GK/FS	GK/FS	GK/FS
**Before completing this checklist, consider:**							
Is the paper relevant to key question?	Yes	yes	yes	yes	yes	yes	yes
**Section 1: INTERNAL VALIDITY**							
The study addresses an appropriate and clearly focused question	yes	yes	yes	yes	yes	yes	yes
The assignment of subjects to treatment groups is randomized	Yes	yes	yes	yes	yes	yes	Yes, in the original study ENESTnd trial (Hughes TP, Saglio G, Kantarjian HM, *et al*. Early molecular response predicts outcomes in patients with chronic myeloid leukemia in chronic phase treated with frontline nilotinib or imatinib. Blood. 2013 doi: 10.1182/blood-2013-06-510396)
An adequate concealment method is used	Yes	yes	yes	yes	yes	yes	Yes
The design keeps subjects and investigators ‘blind’ about treatment allocation	Can’t say	Can’t say	Can’t say	Can’t say	yes	no	Can’t say
The treatment and control groups are similar at the start of the trial	yes	yes	yes	yes	yes	yes	Can’t say
The only difference between groups is the treatment under investigation	no	yes	yes	no	Can’t say	no	No
All relevant outcomes are measured in a standard, valid and reliable way	yes	yes	yes	yes	yes	yes	Yes
What percentage of the individuals or clusters recruited into each treatment arm of the study dropped out before the study was completed	Within a 5-year follow-up, 7 patients progressed to accelerated phase (AP) or blast phase (BP), 4 died because of disease progression, and 3 dropped out of the study because of unsatisfied response or adverse effects.	5 (1.9%) patients in the bosutinib arm and 16 (6.0%) in the imatinib arm had discontinued treatment due to suboptimal response or treatment failure; thus, HRQoL could no longer be assessed longitudinally for these non-responders.	Control group: - Lost to follow up n=4 (Defaulted appointment n=2; Deceased n=2) - Discontinued follow-up n=4 (Withdrawal of consent n=3; TKI interrupted for pregnancy n=1) Intervention group: - Lost to follow up n=4 (Defaulted appointment n=2; Deceased n=1; Transferred to other hospital n=1) - Discontinued intervention n=3 (TKI interrupted due to pregnancy n=1; TKI interrupted due to transplant n=1; TKI withhold due to pancytopenia n=1)	Two of the protocols (Dasatinib and Nilotinib) began enrolling patients before the MDASI‐CML had been developed. After MDASI‐CML was developed, it was added to these two protocols. The MDASICML was part of Ponatinib protocol from the onset of the trial. A total of 219 patients were included and followed over a median of 54 months (range, 8‐112) from the start of therapy. These represent 72% of all patients treated in these studies.	Completion rates were > 80% up to month 9 in the bosutinib arm and up to month 6 in the imatinib arm for the FACT-Leu questionnaire, and up to month 6 in both arms for the EQ-5D questionnaire. At month 12, completion rates were slightly below 80% for both instruments in both study arms (78.9% for bosutinib and 79.3% for imatinib).	In total, 48 of 82 eligible patients (59%) who were approached signed a consent form. Consenters did not differ from refusers by race, ethnicity or sex. As for the retention rate, 41 of 46 consented patients (89%) who remained eligible throughout the study completed both baseline and follow-up assessments. As for the completion rate, 22 of 28 patients (79%) randomized to CBT-TTF received at least 10 sessions. Thus, the study was determined to be acceptable and feasible.	Out of the 846 patients enrolled in the ENESTnd trial, 593 met the inclusion criteria for the post-hoc analyses about HRQoL; among these 593 patients, 33.4% were treated with nilotinib, 34.1% with nilotinib and 32.5% with imatinib.
All the subjects are analyzed in the groups to which they were randomly allocated (often referred to as intention to treat analysis)	can't say (not specified how many males/females, mean±sd age and educational level in each group)	Can’t say	Control group: Analysed (n=64) Intervention group: Analysed (n=65)	Can’t say	Can’t say	On average, patients had been diagnosed with CML 5.2 years previously and had been on their current TKI for 2.5 years. A plurality of patients was receiving dasatinib (36%), which was followed by nilotinib (25%), bosutinib (20%), imatinib (12%), and ponatinib (7%).	Out of the 846 patients enrolled in the ENESTnd trial, 593 met the inclusion criteria for the post-hoc analyses about HRQoL; among these 593 patients, 33.4% were treated with nilotinib, 34.1% with nilotinib and 32.5% with imatinib.
Where the study is carried out at more than one site, results are comparable for all sites	Does not apply	yes	yes	Does not apply	Does not apply	Does not apply	Does not apply
**SECTION 2: OVERALL ASSESMENT OF THE STYDY**							
How well was the study done to minimize bias? Code as follows: “high quality”, “Acceptable”, “Low quality”, “Unacceptable-reject”	High quality	High quality	acceptable	acceptable	High quality	High quality	acceptable
Taking into account clinical considerations, your evaluation of the methodology used, and the statistical power of the study, are you certain that the overall effect is due to the study intervention?	yes	yes	yes	yes	yes	yes	Yes
Are the results of this study directly applicable to the patient group targeted by this guideline?	yes	yes	yes	yes	yes	yes	Yes
Notes. Summarize the authors’ conclusions. Add any comments on your own assessment of the study, and the extent to which it answers your question and mention any areas of uncertainty raised above	Achieving optimal response by 12 months was not only associated with longer survival and lower rates of treatment failure and disease progression, but also with better HRQoL in newly diagnosed patients with CML on front-line TKI. Further studies with larger sample sizes are required to confirm these findings. Marked differences were noted on some HRQoL subscales by demographic or clinical characteristics at baseline, including SF by gender, MH and MCS by level of education, and GH by TKI used. However, each subscale score of HRQoL at baseline between patients achieving optimal response or not at 3, 6, or 12 months during TKI therapy was similar. During the 5-year follow-up period, there was no difference on each subscale score of the HRQoL profile, PCS scores, and MCS scores between patients receiving nilotinib and those receiving imatinib	Mostly of FACT-Leu domains (except social well-being and physical well-being) demonstrated a significant relationship with MR. Results showed variable impact of clinical improvement on different dimensions of HRQoL. For patients who achieved deep MR, emotional well-being and leukemia-specific domains showed the greatest improvement, with medium differences in effect sizes, whereas social wellbeing and physical well-being had the weakest relationship with MR.	The results of the present study demonstrated that the MMS improved the HRQoL score on satisfaction with care and information, as well as the score on satisfaction with social life.	Over time, the effect, real or perceived, of the medication causing or contributing to the HRQoL measures becomes more relevant as the disease achieves optimal control and is not likely to contribute in a meaningful way to the presence of these Symptoms. It is possible to speculate that some of the peaks that occur later may represent a transition from these early stages, to one where the stress caused by the initial diagnosis is relived at least in part as patients appreciate the good response to therapy, and some of the low-grade but chronic adverse events accompanying therapy with TKIs become more evident.	Objective assessment of PROs is becoming increasingly important in CML clinical trials since HRQoL can influence treatment decision-making by physicians and patients, and findings provide valuable evidence regarding the effect of TKIs treatment on HRQoL in this patient population.	This pilot study suggests that internet assisted CBT-TTF is a promising intervention to improve fatigue due to targeted therapies and overall HRQoL of people with CML. Additional research is needed to confirm these findings in larger samples and among other patient populations treated with targeted therapy (i.e.: lung cancer and renal cell carcinoma).	The post-hoc analysis of data from the ENESTnd trial data indicated that certain classes of low-grade AEs significantly impaired the HRQoL of patients with CML who were treated with nilotinib or imatinib, and that all these AEs (except psychiatric disorders) were less frequent in patients treated with nilotinib compared to those treated with imatinib. The impact of low-grade AEs on HRQoL should be taken into account, along with other factors, when selecting the optimal treatment for patients newly diagnosed with CML.

MDASI-CML: MD Anderson Symtom Inventory, Cleeland *et al*. 2000 [...]; SF: Social Functioning; MH: Mental Health; MCS: Mental Component Summary; GH: General Health; PCS: Physical Component Summary; MR: Molecular Response; MMS: Medication Management Service; PROs: Patients-Reported Outcomes; CBT-TTF: Cognitive Behavioral Therapy for Targeted Therapy–Related Fatigue; AEs: Adverse Events.

**Table 2:** Characteristics of the RCTs using TKIs or TKIs plus an add-on treatment and their effects on HRQOL.

**STUDY**	**COUNTRY**	**RCT I ARM TREATMENT** **(N subjects)**	**RCT II ARM TREATMENT** **(N subjects)**	**RCT III ARM TREATMENT** **(N subjects)**	**OVERALL SAMPLE SIZE**	**RCT DURATION** **(total)**	**Add-On Treatment**	**HRQoL PRIMARY OUTCOME**	**EFFECTS ON** **HRQoL** **(Main Findings)**	**Effects ON HRQoL**
Cortes *et al*. 2019 [42]	Multicenter (US, EU)	Imatinib: 400 mg/daily (N 241)	Bosutinib: 400 mg/daily (N 246)		N 536 (N 487: 12 months mITT)	5 years	No	No	At the baseline, FACT-Leu combined and subscale scores were similar in the bosutinib and imatinib arms; after 1 year, all scores demonstrated improvement or maintenance of health-related quality of life (HRQoL) in both treatment arms. Functional health status, as measured by EQ-5D, also demonstrated improvement or maintenance with bosutinib and imatinib after 1 year.	Changes in FACTLeu and EQ-5D scores from baseline values after 12 months of treatment: Bosutinib (*n *= 190) [Mean Δ (SD); *p*] FACT-Leu: Physical well-being [−0.4 (4.5); 0. 2789] Social well-being [−0.2 (5.0); 0. 5366] Emotional well-being [0.9 (3.8); 0.00^13^] Functional well-being [0.8 (5.8); 0.05^45^] Leukemia subscale [1.4 (8.6); 0.0222] FACT-G total [1.2 (13.3); 0.2322] FACT-Leu total [2.6 (20.1); 0.07^62^] TOI FACT-Leu [1.9 (16.1); 0.10^15^] EQ-5D: Utility [−0.001 (0.217); 0.9412] VAS [4.4 (18.3); 0.00^14^] Imatinib (*n *= 189) [Mean Δ (SD); *p*] FACT-Leu: Physical well-being [0.3 (4.4); 0.4217] Social well-being [−0.2 (5.1); 0.5870] Emotional well-being [1.5 (3.7); < 0.000^1^] Functional well-being [0.8 (5.3); 0.0426] Leukemia subscale [2.4 (8.3); 0.000^1^] FACT-G total [2.4 (12.3); 0.0088] FACT-Leu total [4.7 (18.5); 0.000^6^] TOI FACT-Leu [3.4 (14.5); 0.00^16^] EQ-5D: Utility [0.039 (0.175); 0.00^26^] VAS [9.3 (21.7); < 0.000^1^]
Brümmendorf *et al*. 2020 [43]	Multicenter (US, EU)	Imatinib: 400 mg/daily (N 241)	Bosutinib: 400 mg/daily (N 246)		N 536 (N 487: 12 months mITT)	5 years	No	Yes (in association with MR)	The majority of FACT-Leu domains (with the exception of social well-being and physical well-being) demonstrated a significant relationship with MR (p < 0.05). For patients who achieved the highest MR, emotional well-being and leukemia-specific domains showed the greatest improvement, with medium differences in effect sizes, whereas social wellbeing and physical well-being had the weakest relationship with MR.	A (standardized) effect size of 0.2 is considered small (i.e., the difference in means being 0.2 baseline standard deviation units), 0.5 medium, and 0.8 large; a value of ~ 0.1 is trivial; midpoints between values of 0.1, 0.2, 0.5, and 0.8 were used to create categorization intervals for effect size: MR had the most robust relationships with emotional well-being and leukemia-specific scores, showing medium, small, and trivial differences associated with MR5 (Deep MR), MMR (major molecular response), and MR1, respectively. Differences in estimated mean FACT-Leu total and TOI-FACT-Leu scores were small for MR5 and MMR and trivial for MR1. The effect size of the difference in FACT-Leu total score corresponding to MR5 versus MR1 was 0.24, which can be interpreted as small. For FACT-G total and functional well-being scores, differences associated with MR5 were small, and those associated with MMR and MR1 were trivial. MR had the weakest relationships with physical well-being and social well-being, where all differences were considered trivial.
Zulbaran‐Rojas *et al*. 2018 [47]	US	Dasatinib: 100 mg/day (N 104)	Nilotinib: 400 mg twice/daily (N 82)	Ponatinib: 30-45 mg/daily (N 33)	N 219	2 years	No	Yes	Longitudinal analysis on MDASI-CML showed relatively stable Symptom severity scores over time. Fatigue was the most common Symptom in all three cohorts, both prior to the start of therapy and during therapy, including after achievement of deep molecular remission. Work was the most affected component of daily living. Overall patients tolerated therapy well with improvement of their Symptoms from baseline, with few dose reductions related to toxicity or Symptomatology. Although 31% of the patients who completed MDASI‐CML achieved complete molecular remission by 24 months of treatment, nearly 90% experienced persistent mild Symptoms.	There was no significant difference for fatigue mean scores between the three cohorts (*P *> 0.05), but ponatinib had the highest numerical scores in the majority of Symptoms by 24 months. When comparing mean scores between cohorts, patients on ponatinib had higher significant scores of disturbed sleep (*P *= 0.02) than patients on dasatinib, and of malaise (*P *= 0.0113), swelling of extremities (*P *= 0.0003), pain (*P *= 0.0086), shortness of breath (*P *= 0.010), and WAW (*P *= 0.0285) than patients on nilotinib. In addition, skin rash (*P *< 0.0001 vs dasatinib, *P *= 0.0012 vs nilotinib), muscle cramps (*P *= 0.0015 vs dasatinib, *P *= 0.0008 vs nilotinib), dry mouth (*P *< 0.0001), distress (*P *= 0.008 vs dasatinib, *P *= 0.028 vs nilotinib), and REM (*P *= 0.0008) scores were significantly higher with ponatinib compared to both dasatinib and nilotinib cohorts Patients on nilotinib had significant higher scores of disturbed sleep (*P *= 0.026), diarrhea (*P *= 0.009), and swelling of extremities (*P *= 0.02) than patients on dasatinib, while pain had a higher score among patients on dasatinib (*P *= 0.03) compared to those on nilotinib. Importantly, the majority of patients who completed MDASI‐CML experienced persistent mild Symptoms at each time point.
Yu *et al*. 2018 [45]	China	Imatinib 400 mg/daily (N 31)	Nilotinib 300 mg twice/daily (28)		N 59	5 years	No	Yes	Achieving optimal MR after 1 year of treatments was associated with better role limitations due to physical health problems and emotional problems. It was the sole factor associated with significantly improving HRQoL physical dimension over time. Achieving optimal MR at 6 months had high probability of better physical and social functioning, and reduced role limitations due to emotional problems. Furthermore, age < 40 years, female gender, and higher level of education were factors also associated with better HRQoL subscale scores. However, optimal response at 3 months had no impact on HRQoL profile.	Multivariate analyses showed that achievement of optimal response at 6 months was associated with a tendency of having high PF (P = .0674), SF (P = 0.0571), and RE (P = 0.0916) scores, while achieving optimal response at 12 months was associated with markedly higher RP (P = 0.0019) and RE (P = 0.0110) scores. In addition, age < 40 years was associated with better PF (P = 0.0005), PCS (P = 0.0209), SF (P = 0.0008), and RE (P = 0.0493) scores; female gender was associated with better SF (P = 0.0370) and RE (P = 0.0315) scores; and a higher level of education was associated with better BP (P = 0.0467). Response at 3 months and the TKI used (imatinib or nilotinib) did not show any impact on the HRQoL outcomes during TKI therapy. Multivariate analyses showed that PCS scores were constant throughout the treatment (P = 0.9913), while MCS scores showed a tendency toward gradual increase (P = 0.0611) with continuation of treatment; however, achieving optimal response at 12 months was the sole factor associated with a significant improvement in PCS scores over time (P = .0160)
Guérin *et al*. 2014 [44]	Multicenter (US)	Nilotinib: 300 mg twice/daily (N 282)	Nilotinib: 400 mg twice/daily (N 281)	Imatinib: 400 mg/daily (N 283)	N 846	2 years	No	Yes (post-hoc analysis)	Five low-grade adverse events (AEs) categories (gastrointestinal, blood and lymphatic systems, musculoskeletal, psychiatric Symptoms, general disorders and administration site conditions) significantly impaired at least one of the HRQoL scores. The incidence rate of these five AEs was significantly lower for nilotinib than imatinib or not different between the two drugs. The AEs categories with lower incidence for both nilotinib 300 mg twice/daily and 400 mg twice/daily versus imatinib 400 mg daily were gastrointestinal, blood and lymphatic system and musculoskeletal Symptoms; nilotinib 300 mg twice/daily had lower incidence than imatinib for general disorders.	Among the 2.566 FACT-Leu assessments included in the analyses, 58.5% showed an improvement from baseline (mean improvement +15.7 ± 13.6 SD), 39.9% showed a deterioration from baseline (mean deterioration –14.1 ± 12.9 SD), and 1.6% showed no change from baseline. The respective percentages for SF-36-Physical were 53.5% (mean improvement +7.5 ± 6.2), 46.1% (mean deterioration –6.1 ± 5.3) and 0.4% (no change) respectively, while for SF-36-Mental they were 63.1% (mean improvement +9.6 ± 8.2), 36.5% (mean deterioration –6.7 ± 6.5) and 0.4% (no change). Overall, 250 (9.7%) of the 2.566 FACT-Leu assessments and 747 (30.8%) of the 2.423 SF-36 assessments were preceded by a low-grade AE during their corresponding 7- and 28-day recall periods.
Jim *et al*. 2020 [48]	US	TKI therapy + internet-assisted CBT-TTF (N 29)	TKI therapy + WLC (N 15)		N 44	18 weeks	Yes	No	Among the patients treated with TKIs assigned to CBT-TTF, 79% completed the intervention. Intent-to- treat analyses showed that patients assigned to CBT-TTF demonstrated greater improvements in fatigue and overall HRQoL than those assigned to WLC. More patients randomized to CBT-TTF than WLC demonstrated clinically significant improvements in fatigue and H-QoL.	Effect size magnitudes were interpreted as follows: small (0.2–0.5), medium (0.5–0.8), or large (>0.8). As for the primary outcome of fatigue severity, participants randomized to CBT-TTF demonstrated significantly greater improvements than those randomized to WLC (P < .001).
Jim *et al*. 2020 [48]	US	TKI therapy + internet-assisted CBT-TTF (N 29)	TKI therapy + WLC (N 15)		N 44	18 weeks	Yes	No	Among the patients treated with TKIs assigned to CBT-TTF, 79% completed the intervention. Intent-to- treat analyses showed that patients assigned to CBT-TTF demonstrated greater improvements in fatigue and overall HRQoL than those assigned to WLC. More patients randomized to CBT-TTF than WLC demonstrated clinically significant improvements in fatigue and H-QoL.	The effect size for fatigue severity was large (d = 1.07). As for secondary outcomes, more CBT-TTF participants (85%) experienced clinically significant improvements in fatigue than WLC participants (29%; P < .001). Significantly greater improvements in the CBT-TTF group were also observed for overall quality of life (P = .005). The effect size for quality of life was also large (d = 1.15). More CBT-TTF participants (88%) experienced clinically significant improvements in quality of life than WLC participants (54%; P < .016). As for the quality-of-life subscales, greater improvements in the CBT-TTF group than the WLC group were observed for FWB (d = 1.06; P = .003) and EWB (d = 1.12; P < .001) but not PWB (d = 0.45; P = .151) or SWB (d = 0.60; P = .800).
Tan *et al*. 2019 [46]	Malaysia	TKI therapy + medication management service-MMS (N 65)	TKI therapy + usual care (usual pharmacy services) (N 64)		N 129	1 year	yes	No	Among the patients treated with TKIs, 3 out of 20 subscales of EORTC-QLQ30-CML24 (financial difficulties, satisfaction with care and information, satisfaction with social life were significantly better in the MMS group than in the usual-care group after 6 months from the baseline. Other 3 subscale (cognitive functioning, insomnia and impact on worry and mood) was also significantly improved after 12 months in the MMS arm than in the usual-care arm.	At 6th month, participants in the MMS arm had a significantly improved mean HRQoL score than participants in control arm in the three subscales of EORTC QLQ-30_CML24, including financial difficulties (13.33 ± 10.75 vs 22.78 ± 21.59; p = 0.016), satisfaction with care and information (85.83 ± 18.62 vs 56.67 ± 30.41; p < 0.001) and satisfaction with social life (76.67 ± 22.38 vs 57.78 ± 34.10; p = 0.002).
Tan *et al*. 2019 [46]	Malaysia	TKI therapy + medication management service-MMS (N 65)	TKI therapy + usual care (usual pharmacy services) (N 64)		N 129	1 year	yes	No	Among the patients treated with TKIs, 3 out of 20 subscales of EORTC-QLQ30-CML24 (financial difficulties, satisfaction with care and information, satisfaction with social life were significantly better in the MMS group than in the usual-care group after 6 months from the baseline. Other 3 subscale (cognitive functioning, insomnia and impact on worry and mood) was also significantly improved after 12 months in the MMS arm than in the usual-care arm.	At 12th month, the mean score of three other subscales including cognitive functioning, insomnia and impact on worry and mood was also significantly improved in the MMS arm. None of the HRQoL subscales was associated with the development of comorbidities except for pain (OR 9.226; 95% CI 1.219, 17.232; p = 0.024).

mITT: Modified Intention-to-treat; MR: Molecular Response; CBT-TTF: Cognitive-Behavioral Therapy Targeted Therapy-related Fatigue; WLC: Waiting List Control; MMS: Medication Management Service; MDASI-CML: MD Anderson Symptom Inventory; REM: Relations with others, Enjoyment of Life, and Mood; WAW: Work, Activity, and Walk; PF: Physical Functioning; RP: Role Limitation due to Physical Health Problems; BP: Bodily Pain; GH: General Health Perceptions; VT: Vitality; SF: Social Functioning; RE: Role Limitations due to Emotional Problems; MH: Mental Health; PCS: Physical Component Summary; MCS: Mental Component Summary; AE: Adverse Event.

**Table 3:** Instruments used to assess HRQoL in the included RCTs.

**-**	**FACT-leu** Cella *et al*. 2012 [49]	**EuroQoL-5 Dimensions (EQ-5D)** EuroQoL-Group, 1990; Van Reenan & Oppe, 2015; https://euroqol.org/publications/user-guides/ [50, 51]	**MD Anderson Symptom Inventory (MDASI-CML)** Cleeland *et al*. 2000 [52]	**Short Form Health Survey – 36 item (SF-36)** Ware *et al*. 1992 [53]	**European Organization for Research and Treatment of Cancer (EORTC)_QLQ-30_CML-24** Efficace *et al*. 2014 [54]
**Included RCTs**	-	-	-	-	-
Cortes *et al*. 2019 [42]	X	X			
Brümmendorf *et al*. 2020 [43]	X				
Zulbaran-Rojas *et al*. 2018 [47]			X		
Yu *et al*. 2018 [45]				X	
Guérin *et al*. 2014 [44]	X			X	
Jim *et al*. 2020 [48]	X (only the FACT-G scale)				
Tan *et al*. 2019 [46]					X

## SUPPLEMENTARY MATERIAL

PRISMA checklist is available as supplementary material on the publisher’s website along with the published article.

## References

[1] Jabbour E., Kantarjian H. (2020). Chronic myeloid leukemia: 2020 update on diagnosis, therapy and monitoring.. Am. J. Hematol..

[2] Faderl S., Talpaz M., Estrov Z., O’Brien S., Kurzrock R., Kantarjian H.M. (1999). The biology of chronic myeloid leukemia.. N. Engl. J. Med..

[3] Hochhaus A., Baccarani M., Silver R.T., Schiffer C., Apperley J.F., Cervantes F., Clark R.E., Cortes J.E., Deininger M.W., Guilhot F., Hjorth-Hansen H., Hughes T.P., Janssen J.J.W.M., Kantarjian H.M., Kim D.W., Larson R.A., Lipton J.H., Mahon F.X., Mayer J., Nicolini F., Niederwieser D., Pane F., Radich J.P., Rea D., Richter J., Rosti G., Rousselot P., Saglio G., Saußele S., Soverini S., Steegmann J.L., Turkina A., Zaritskey A., Hehlmann R. (2020). European LeukemiaNet 2020 recommendations for treating chronic myeloid leukemia.. Leukemia.

[4] Baccarani M., Abruzzese E., Accurso V., Albano F., Annunziata M., Barulli S., Beltrami G., Bergamaschi M., Binotto G., Bocchia M., Caocci G., Capodanno I., Cavazzini F., Cedrone M., Cerrano M., Crugnola M., D’Adda M., Elena C., Fava C., Fazi P., Fozza C., Galimberti S., Giai V., Gozzini A., Gugliotta G., Iurlo A., La Barba G., Levato L., Lucchesi A., Luciano L., Lunghi F., Lunghi M., Malagola M., Marasca R., Martino B., Melpignano A., Miggiano M.C., Montefusco E., Musolino C., Palmieri F., Pregno P., Rapezzi D., Rege-Cambrin G., Rupoli S., Salvucci M., Sancetta R., Sica S., Spadano R., Stagno F., Tiribelli M., Tomassetti S., Trabacchi E., Bonifacio M., Breccia M., Castagnetti F., Pane F., Russo D., Saglio G., Soverini S., Vigneri P., Rosti G. (2019). Managing chronic myeloid leukemia for treatment-free remission: a proposal from the GIMEMA CML WP.. Blood Adv..

[5] Etienne G, Milpied B, Réa D, Rigal-Huguet F, Tulliez M, Nicolini FE (2010). French Intergroup of CML (Fi-LMC group). Recommandations du groupe Fi-LMC pour la gestion des effets indésirables du traitement par nilotinib (Tasigna) au cours de la leucémie myéloïde chronique [Guidelines for the management of nilotinib (Tasigna)-induced side effects in chronic myelogenous leukemia: recommendations of French Intergroup of CML (Fi-LMC group)].. Bull Cancer.

[6] le Coutre P., Ottmann O.G., Giles F., Kim D.W., Cortes J., Gattermann N., Apperley J.F., Larson R.A., Abruzzese E., O’Brien S.G., Kuliczkowski K., Hochhaus A., Mahon F.X., Saglio G., Gobbi M., Kwong Y.L., Baccarani M., Hughes T., Martinelli G., Radich J.P., Zheng M., Shou Y., Kantarjian H. (2008). Nilotinib (formerly AMN107), a highly selective BCR-ABL tyrosine kinase inhibitor, is active in patients with imatinib-resistant or -intolerant accelerated-phase chronic myelogenous leukemia.. Blood.

[7] Caocci G., Mulas O., Abruzzese E., Luciano L., Iurlo A., Attolico I., Castagnetti F., Galimberti S., Sgherza N., Bonifacio M., Annunziata M., Gozzini A., Orlandi E.M., Stagno F., Binotto G., Pregno P., Fozza C., Trawinska M.M., De Gregorio F., Cattaneo D., Albano F., Gugliotta G., Baratè C., Scaffidi L., Elena C., Pirillo F., Scalzulli E., La Nasa G., Foà R., Breccia M. (2019). Arterial occlusive events in chronic myeloid leukemia patients treated with ponatinib in the real-life practice are predicted by the Systematic Coronary Risk Evaluation (SCORE) chart.. Hematol. Oncol..

[8] Kekäle M., Peltoniemi M., Airaksinen M. (2015). Patient-reported adverse drug reactions and their influence on adherence and quality of life of chronic myeloid leukemia patients on per oral tyrosine kinase inhibitor treatment.. Patient Prefer. Adherence.

[9] Caocci G., Mulas O., Bonifacio M., Abruzzese E., Galimberti S., Orlandi E.M., Iurlo A., Annunziata M., Luciano L., Castagnetti F., Gozzini A., Stagno F., Binotto G., Pregno P., Albano F., Martino B., Fozza C., Scaffidi L., Trawinska M.M., Baratè C., Elena C., Cattaneo D., Scalzulli E., La Nasa G., Foà R., Breccia M. (2019). Recurrent arterial occlusive events in patients with chronic myeloid leukemia treated with second- and third-generation tyrosine kinase inhibitors and role of secondary prevention.. Int. J. Cardiol..

[10] Caocci G., Atzeni S., Orrù N., Azzena L., Martorana L., Littera R., Ledda A., La Nasa G. (2008). Gynecomastia in a male after dasatinib treatment for chronic myeloid leukemia.. Leukemia.

[11] Cella D., Nowinski C.J., Frankfurt O. (2014). The impact of Symptom burden on patient quality of life in chronic myeloid leukemia.. Oncology.

[12] Marin D., Bazeos A., Mahon F.X., Eliasson L., Milojkovic D., Bua M., Apperley J.F., Szydlo R., Desai R., Kozlowski K., Paliompeis C., Latham V., Foroni L., Molimard M., Reid A., Rezvani K., de Lavallade H., Guallar C., Goldman J., Khorashad J.S. (2010). Adherence is the critical factor for achieving molecular responses in patients with chronic myeloid leukemia who achieve complete cytogenetic responses on imatinib.. J. Clin. Oncol..

[13] Kekäle M., Söderlund T., Koskenvesa P., Talvensaari K., Airaksinen M. (2016). Impact of tailored patient education on adherence of patients with chronic myeloid leukaemia to tyrosine kinase inhibitors: a randomized multicentre intervention study.. J. Adv. Nurs..

[14] Leader A., Benyamini N., Gafter-Gvili A., Dreyer J., Calvarysky B., Amitai A., Yarchovsky-Dolberg O., Sharf G., Tousset E., Caspi O., Ellis M., Levi I., De Geest S., Raanani P. (2018). Effect of Adherence-enhancing Interventions on Adherence to Tyrosine Kinase Inhibitor Treatment in Chronic Myeloid Leukemia (TAKE-IT): A Quasi-experimental Pre-Post Intervention Multicenter Pilot Study.. Clin. Lymphoma Myeloma Leuk..

[15] Moulin S.M., Eutrópio F.J., Souza J.O., Busato F.O., Olivieri D.N., Tadokoro C.E. (2017). The role of clinical pharmacists in treatment adherence: fast impact in suppression of chronic myeloid leukemia development and Symptoms.. Support. Care Cancer.

[16] Johnson J.R., Temple R. (1985). Food and Drug Administration requirements for approval of new anticancer drugs.. Cancer Treat. Rep..

[17] U.S. Department of Health and Human Services FDA Center for Drug Evaluation and Research, U.S. Department of Health and Human Services FDA Center for Biologics Evaluation and Research (2006). U.S. Department of Health and Human Services FDA Center for Devices and Radiological Health. Guidance for industry: patient-reported outcome measures: use in medical product development to support labeling claims: draft guidance.. Health Qual. Life Outcomes.

[18] Mantovani G., Astara G., Lampis B., Bianchi A., Curreli L., Orrù W., Carta M.G., Carpiniello B., Contu P., Rudas N. (1996). Evaluation by multidimensional instruments of health-related quality of life of elderly cancer patients undergoing three different “psychosocial” treatment approaches. A randomized clinical trial.. Support. Care Cancer.

[19] Giesinger J.M., La Nasa G., Sparano F. (2020). Health-related quality of life assessment in patients with myelodysplastic syndromes: evidence from randomized controlled trials.. Clin. Pract. Epidemiol. Ment. Health.

[20] Cannella L., Caocci G., Jacobs M., Vignetti M., Mandelli F., Efficace F. (2015). Health-related quality of life and Symptom assessment in randomized controlled trials of patients with leukemia and myelodysplastic syndromes: What have we learned?. Crit. Rev. Oncol. Hematol..

[21] Efficace F., Gaidano G., Sprangers M., Cottone F., Breccia M., Voso M.T., Caocci G., Stauder R., Di Tucci A.A., Sanpaolo G., Selleslag D., Angelucci E., Platzbecker U., Mandelli F. (2014). Preference for involvement in treatment decisions and request for prognostic information in newly diagnosed patients with higher-risk myelodysplastic syndromes.. Ann. Oncol..

[22] Caocci G., Voso M.T., Angelucci E., Stauder R., Cottone F., Abel G., Nguyen K., Platzbecker U., Beyne-Rauzy O., Gaidano G., Invernizzi R., Molica S., Criscuolo M., Breccia M., Lübbert M., Sanpaolo G., Buccisano F., Ricco A., Palumbo G.A., Niscola P., Zhang H., Fenu S., La Nasa G., Mandelli F., Efficace F. (2015). Accuracy of physician assessment of treatment preferences and health status in elderly patients with higher-risk myelodysplastic syndromes.. Leuk. Res..

[23] Björkholm M., Ohm L., Eloranta S., Derolf A., Hultcrantz M., Sjöberg J., Andersson T., Höglund M., Richter J., Landgren O., Kristinsson S.Y., Dickman P.W. (2011). Success story of targeted therapy in chronic myeloid leukemia: a population-based study of patients diagnosed in Sweden from 1973 to 2008.. J. Clin. Oncol..

[24] Flynn K.E., Atallah E. (2016). Quality of life and long-term therapy in patients with chronic myeloid leukemia.. Curr. Hematol. Malig. Rep..

[25] Efficace F., Cardoni A., Cottone F., Vignetti M., Mandelli F. (2013). Tyrosine-kinase inhibitors and patient-reported outcomes in chronic myeloid leukemia: a systematic review.. Leuk. Res..

[26] Efficace F., Cocks K., Breccia M., Sprangers M., Meyers C.A., Vignetti M., Baccarani M., Mandelli F., GIMEMA and EORTC Quality of Life Group (2012). Time for a new era in the evaluation of targeted therapies for patients with chronic myeloid leukemia: inclusion of quality of life and other patient-reported outcomes.. Crit. Rev. Oncol. Hematol..

[27] Leplège A., Hunt S. (1997). The problem of quality of life in medicine.. JAMA.

[28] Karimi M., Brazier J. (2016). Health, health-related quality of life, and quality of life: What is the difference?. PharmacoEconomics.

[29] Hays R.D., Reeve B.B., Killewo J., Heggenhougen H.K., Quah S.R. (2010). Epidemiology and Demography in Public Health..

[30] Torrance G.W. (1987). Utility approach to measuring health-related quality of life.. J. Chronic Dis..

[31] Sancassiani F., Carmassi C., Romano F., Balestrieri M., Caraci F., Di Sciascio G., Drago F., Faravelli C., Hardoy M.C., Moro M.F., Roncone R., Preti A., Dell’Osso L. (2019). Impairment of quality of life associated with lifetime diagnosis of post-traumatic stress disorder in women - A national survey in Italy.. Clin. Pract. Epidemiol. Ment. Health.

[32] Carta M.G., Aguglia E., Caraci F., Dell’Osso L., Di Sciascio G., Drago F., Del Giudice E., Faravelli C., Hardoy M.C., Lecca M.E., Moro M.F., Calò S., Casacchia M., Angermeyer M., Balestrieri M. (2012). Quality of life and urban / rural living: Preliminary results of a community survey in Italy.. Clin. Pract. Epidemiol. Ment. Health.

[33] Carta M.G., Moro M.F., Lorefice L., Picardi A., Trincas G., Fenu G., Cocco E., Floris F., Bessonov D., Akiskal H.S., Marrosu M.G. (2014). Multiple sclerosis and bipolar disorders: the burden of comorbidity and its consequences on quality of life.. J. Affect. Disord..

[34] Carta MG, Conti A, Lecca F (2015). The burden of depressive and bipolar disorders in celiac disease.. Clin Pract Epidemiol Ment Health.

[35] Carta M.G., Moro M.F., Pinna F.L., Testa G., Cacace E., Ruggiero V., Piras M., Romano F., Minerba L., Machado S., Freire R.C., Nardi A.E., Sancassiani F. (2018). The impact of fibromyalgia syndrome and the role of comorbidity with mood and post-traumatic stress disorder in worsening the quality of life.. Int. J. Soc. Psychiatry.

[36] Carta MG, Lecca ME, Saba L (2015). Patients with carotid atherosclerosis who underwent or did not undergo carotid endarterectomy: outcome on mood, cognition and quality of life.. BMC Psychiatry.

[37] Carta MG, Sorbello O, Moro MF (2012). Bipolar disorders and Wilson's disease.. BMC Psychiatry.

[38] La Nasa G., Caocci G., Morelli E., Massa E., Farci A., Deiana L., Pintus E., Scartozzi M., Sancassiani F. (2020). Health related quality of life in patients with onco-hematological diseases.. Clin. Pract. Epidemiol. Ment. Health.

[39] Aviles Gonzalez C.I., Caocci G., Angermeyer M. (2020). The attributable burden to solid cancer in worsening Health-related Quality of Life is lower than in disorders with a less severe prognosis but it gets worse by depression.. Clin. Pract. Epidemiol. Ment. Health.

[40] Moher D., Shamseer L., Clarke M., Ghersi D., Liberati A., Petticrew M., Shekelle P., Stewart L.A. (2015). PRISMA-P Group. Preferred reporting items for systematic review and meta-analysis protocols (PRISMA-P) statement.. Syst. Rev..

[41] Cortes J.E., Gambacorti-Passerini C., Deininger M.W., Mauro M.J., Chuah C., Kim D.W., Dyagil I., Glushko N., Milojkovic D., le Coutre P., Garcia-Gutierrez V., Reilly L., Jeynes-Ellis A., Leip E., Bardy-Bouxin N., Hochhaus A., Brümmendorf T.H. (2018). Bosutinib *versus* Imatinib for newly diagnosed chronic myeloid leukemia: Results from the randomized bfore trial.. J. Clin. Oncol..

[42] Cortes J.E., Gambacorti-Passerini C., Deininger M.W., Mauro M.J., Chuah C., Kim D.W., Milojkovic D., le Coutre P., Garcia-Gutierrez V., Crescenzo R., Mamolo C., Reisman A., Hochhaus A., Brümmendorf T.H. (2019). Patient-reported outcomes in the phase 3 BFORE trial of bosutinib *versus* imatinib for newly diagnosed chronic phase chronic myeloid leukemia.. J. Cancer Res. Clin. Oncol..

[43] Brümmendorf T.H., Gambacorti-Passerini C., Bushmakin A.G., Cappelleri J.C., Viqueira A., Reisman A., Isfort S., Mamolo C. (2020). Relationship between molecular response and quality of life with bosutinib or imatinib for chronic myeloid leukemia.. Ann. Hematol..

[44] Guérin A., Chen L., Ionescu-Ittu R., Marynchenko M., Nitulescu R., Hiscock R., Keir C., Wu E.Q. (2014). Impact of low-grade adverse events on health-related quality of life in adult patients receiving imatinib or nilotinib for newly diagnosed Philadelphia chromosome positive chronic myelogenous leukemia in chronic phase.. Curr. Med. Res. Opin..

[45] Yu L., Wang H., Milijkovic D., Huang X., Jiang Q. (2018). Achieving optimal response at 12 months is associated with a better health-related quality of life in patients with chronic myeloid leukemia: a prospective, longitudinal, single center study.. BMC Cancer.

[46] Tan B.K., Chua S.S., Chen L.C., Chang K.M., Balashanker S., Bee P.C. (2020). Efficacy of a medication management service in improving adherence to tyrosine kinase inhibitors and clinical outcomes of patients with chronic myeloid leukaemia: a randomised controlled trial.. Support. Care Cancer.

[47] Zulbaran-Rojas A., Lin H.K., Shi Q., Williams L.A., George B., Garcia-Manero G., Jabbour E., O’Brien S., Ravandi F., Wierda W., Estrov Z., Borthakur G., Kadia T., Cleeland C., Cortes J.E., Kantarjian H. (2018). A prospective analysis of Symptom burden for patients with chronic myeloid leukemia in chronic phase treated with frontline second- and third-generation tyrosine kinase inhibitors.. Cancer Med..

[48] Jim H.S.L., Hyland K.A., Nelson A.M., Pinilla-Ibarz J., Sweet K., Gielissen M., Bulls H., Hoogland A.I., Jacobsen P.B., Knoop H. (2020). Internet-assisted cognitive behavioral intervention for targeted therapy-related fatigue in chronic myeloid leukemia: Results from a pilot randomized trial.. Cancer.

[49] Cella D., Jensen S.E., Webster K., Hongyan D., Lai J.S., Rosen S., Tallman M.S., Yount S. (2012). Measuring health-related quality of life in leukemia: The functional assessment of cancer therapy--leukemia (FACT-Leu) questionnaire.. Value Health.

[50] EuroQol Group (1990). EuroQol--a new facility for the measurement of health-related quality of life.. Health Policy.

[51] van Reenan M., Oppe M. (2015). EQ-5D-3L user guide, v5.1. Basic information on how to use the EQ-5D-3L instrument. EuroQol Research Foundation.. https://euroqol.org/publications/user-guides/.

[52] Cleeland C.S., Mendoza T.R., Wang X.S., Chou C., Harle M.T., Morrissey M., Engstrom M.C. (2000). Assessing Symptom distress in cancer patients: the M.D. Anderson Symptom Inventory.. Cancer.

[53] Ware J.E., Sherbourne C.D. (1992). The MOS 36-item short-form health survey (SF-36). I. Conceptual framework and item selection.. Med. Care.

[54] Efficace F., Baccarani M., Breccia M., Saussele S., Abel G., Caocci G., Guilhot F., Cocks K., Naeem A., Sprangers M., Oerlemans S., Chie W., Castagnetti F., Bombaci F., Sharf G., Cardoni A., Noens L., Pallua S., Salvucci M., Nicolatou-Galitis O., Rosti G., Mandelli F. (2014). International development of an EORTC questionnaire for assessing health-related quality of life in chronic myeloid leukemia patients: the EORTC QLQ-CML24.. Qual. Life Res..

[55] (2011). MedDRA Maintenance and Support Services, International Federation of Pharmaceutical Manufacturers & Associations (IFPMA). Introductory Guide MedDRA Version 17.0. International Conference on Harmonisation (ICH) of Technical Requirements for Registration of Pharmaceuticals for Human Use. https://www.meddra.org/sites/default/files/guidance/file/intguide_17_0_english.pdf.

[56] Efficace F., Cannella L. (2016). The value of quality of life assessment in chronic myeloid leukemia patients receiving tyrosine kinase inhibitors.. Hematology (Am. Soc. Hematol. Educ. Program).

[57] (1995). The World Health Organization Quality of Life assessment (WHOQOL): position paper from the World Health Organization.. Soc. Sci. Med..

[58] Ebrahim S. (1995). Clinical and public health perspectives and applications of health-related quality of life measurement.. Soc. Sci. Med..

[59] Bowling A. (1995). What things are important in people’s lives? A survey of the public’s judgements to inform scales of health related quality of life.. Soc. Sci. Med..

[60] Hall A. (2020). Quality of life and value assessment in health care.. Health Care Anal..

[61] Efficace F., Stagno F., Iurlo A., Breccia M., Cottone F., Bonifacio M., Abruzzese E., Castagnetti F., Caocci G., Crugnola M., Capodanno I., Martino B., Tiribelli M., Patriarca A., Gozzini A., Pregno P., Saussele S., Cascavilla N., Fozza C., Bergamaschi M., Binotto G., Vignetti M., Rosti G. (2020). Health-related quality of life of newly diagnosed chronic myeloid leukemia patients treated with first-line dasatinib *versus* imatinib therapy.. Leukemia.

[62] (2007). Novartis Pharmaceuticals. A Study of Imatinib *versus* Nilotinib in Adult Patients With Newly Diagnosed Philadelphia Chromosome Positive (Ph+) Chronic Myelogenous Leukemia in Chronic Phase (CML-CP). ClinicalTrials.gov.. https://clinicaltrials.gov/ct2/show/NCT00471497.

[63] Trask P.C., Cella D., Besson N., Kelly V., Masszi T., Kim D.W. (2012). Health-related quality of life of bosutinib (SKI-606) in imatinib-resistant or imatinib-intolerant chronic phase chronic myeloid leukemia.. Leuk. Res..

